# Health is beyond genetics: on the integration of lifestyle and environment in real-time for hyper-personalized medicine

**DOI:** 10.3389/fpubh.2024.1522673

**Published:** 2025-01-07

**Authors:** Myles Joshua Toledo Tan, Harishwar Reddy Kasireddy, Alfredo Bayu Satriya, Hezerul Abdul Karim, Nouar AlDahoul

**Affiliations:** ^1^Department of Electrical and Computer Engineering, Herbert Wertheim College of Engineering, University of Florida, Gainesville, FL, United States; ^2^Department of Epidemiology, College of Public Health and Health Professions and College of Medicine, University of Florida, Gainesville, FL, United States; ^3^Biology Program, College of Arts and Sciences, University of St. La Salle, Bacolod, Philippines; ^4^Department of Natural Sciences, College of Arts and Sciences, University of St. La Salle, Bacolod, Philippines; ^5^Department of Chemical Engineering, College of Engineering and Technology, University of St. La Salle, Bacolod, Philippines; ^6^Department of Electronics Engineering, College of Engineering and Technology, University of St. La Salle, Bacolod, Philippines; ^7^Yo-Vivo Corporation, Bacolod, Philippines; ^8^Division of Nephrology, Hypertension and Renal Transplantation – Quantitative Health Section, Department of Medicine, College of Medicine, University of Florida, Gainesville, FL, United States; ^9^Faculty of Engineering, Multimedia University, Cyberjaya, Selangor, Malaysia; ^10^Department of Computer Science, Division of Science, New York University Abu Dhabi, Abu Dhabi, United Arab Emirates

**Keywords:** hyper-personalized medicine, *Healthcare 5.0*, 6G, internet of things, artificial general intelligence, quantum computing, real-time healthcare, smart healthcare

## 1 Introduction

Hyper-personalized medicine represents the cutting edge of healthcare, which aims to tailor treatment and prevention strategies uniquely to each individual. Unlike traditional approaches, which often adopt a one-size-fits-all or even broadly personalized approach based on broad genetic categories, hyper-personalized medicine considers an individual's comprehensive health data by integrating unique biological, genetic, lifestyle, and environmental influences. This method goes beyond simple genetic profiling by recognizing that health outcomes are influenced by complex interactions among our environment, daily routines, and physiological processes and responses.

Central to hyper-personalized medicine is the integration of lifestyle and environmental factors. Lifestyle habits, such as diet (2–8), exercise (9–16), and sleep patterns (5, 17–22), directly impact health. Hence, understanding these factors helps tailor interventions that align with the day-to-day realities of an individual. Environmental factors, such as air quality (23–28), climate (29–36), and exposure to pollutants (37–46), also play significant roles in determining health outcomes. By continuously monitoring and analyzing these elements, healthcare providers can create dynamic health plans that adapt to real-time changes. This would allow for proactive measures and optimized care.

To enable such a complex model of care, advanced technologies like quantum computing, artificial general intelligence (AGI), internet of things (IoT), and 6G connectivity play crucial roles. Quantum computing offers the ability to process vast and intricate datasets, such as those required to model interactions between genetic markers, environmental exposures, and lifestyle choices, with far greater speed and accuracy than classical computing (47–51). AGI, with its adaptive learning capabilities, can analyze and make sense of this data to provide precise, evolving recommendations that change as a patient's environment or lifestyle does (52–55). IoT devices, including wearables and environmental sensors, gather continuous data from individuals, tracking physical activity, biometrics, and environmental conditions like air quality and humidity (56–61). With the advent of 6G connectivity, this data can be seamlessly transferred and processed in real time, enabling instant feedback and intervention (62–67).

Together, these technologies form the backbone of a hyper-personalized healthcare model, which will push beyond traditional medical practices to create a highly responsive and individual-centered approach to health. As these advancements continue to evolve, hyper-personalized medicine has the potential to fundamentally reshape healthcare, offering truly personalized interventions that support long-term health and wellbeing.

## 2 Definition of *Healthcare 5.0*

The European Commission's *Industry 5.0* framework (1), which prioritizes sustainability, human-centered approaches, and resilience, provides an insightful basis for defining a similarly advanced framework in healthcare. This framework aligns naturally with hyper-personalized medicine, as the goal of *Healthcare 5.0* should be to provide not only individualized care but also sustainable, human-centric, and adaptable healthcare systems.

**Figure 1:**
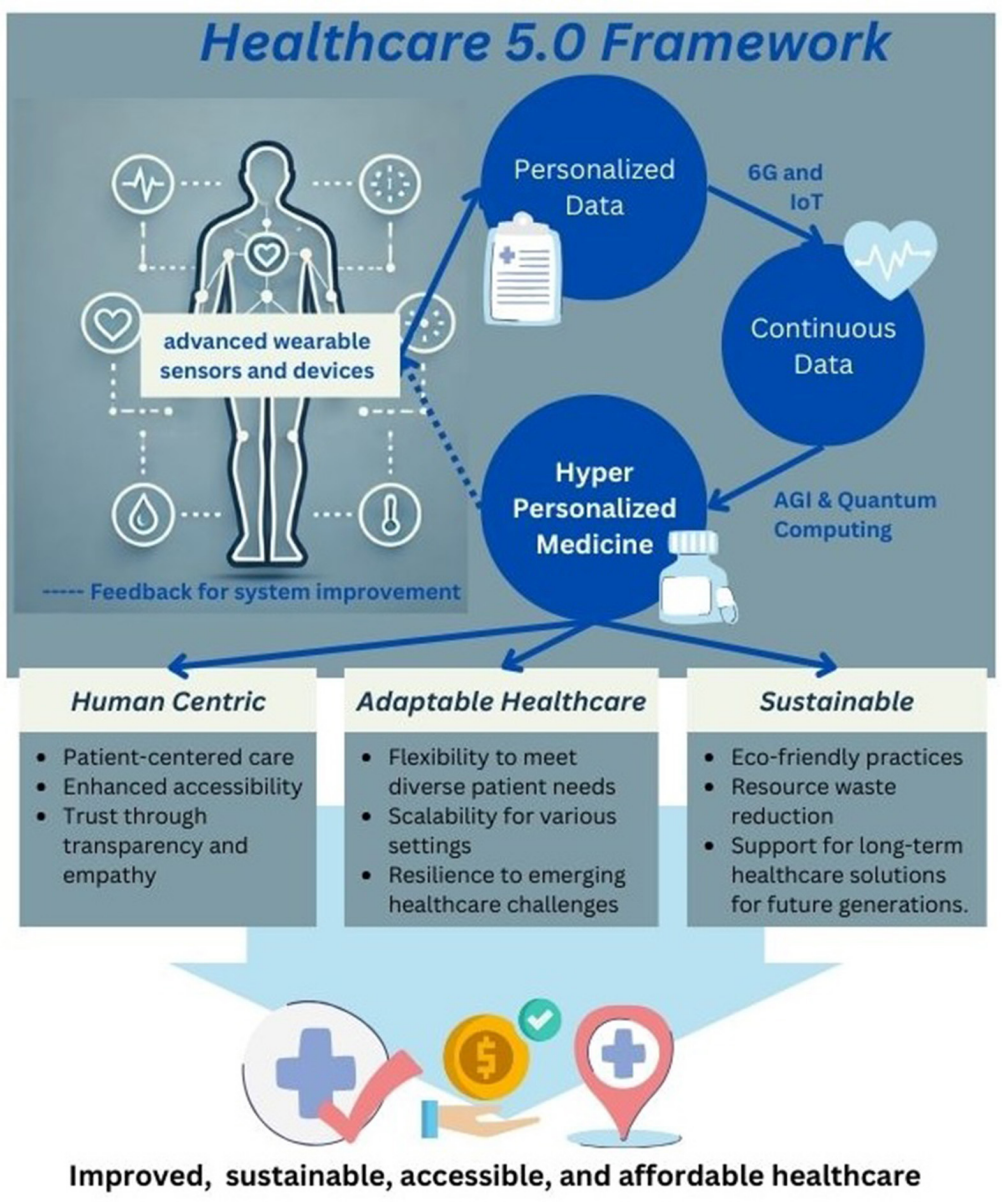
*Healthcare 5.0* framework. The framework illustrates the integration of hyper-personalized medicine through advanced wearable sensors, personalized data, and continuous data streams enabled by 6G, IoT, AGI, and quantum computing. It is built on three pillars: human-centric care (emphasizing patient-centered approaches, transparency, and empathy), adaptable healthcare (ensuring flexibility, scalability, and resilience), and sustainability (promoting eco-friendly practices, resource efficiency, and long-term solutions). This approach aims to achieve improved, sustainable, accessible, and affordable healthcare outcomes.

Hyper-personalized medicine, which tailors treatments and preventive measures based on the unique genetic, lifestyle, and environmental factors of each patient, embodies these principles, as depicted in Figure 1. By focusing on individual needs and integrating real-time data from IoT devices, advanced AI models, and sustainable technology, hyper-personalized medicine enables healthcare systems that prioritize patient wellbeing while adapting to both environmental challenges and individual health needs.

*Healthcare 5.0*, modeled after *Industry 5.0* (1), would thus be defined by its commitment to environmental consciousness (through sustainable resource use and minimized waste), human-centered care (with each patient's unique data guiding treatment), and resilience (enabling healthcare systems to adapt swiftly to changing conditions and global health challenges). This alignment with *Industry 5.0* (1) principles helps ensure that *Healthcare 5.0* does not merely advance medical technology but also builds a health infrastructure that is robust, equitable, and focused on long-term human and planetary wellbeing.

## 3 Lifestyle and environmental factors in health

### 3.1 Effects of lifestyle on individual health

Lifestyle factors, including diet, physical activity, sleep, and stress, play pivotal roles in shaping individual health outcomes. They influence both physical and mental wellbeing. Diet is fundamental to health, particularly in children and adolescents, where high diet quality is associated with better long-term health outcomes and reduced risks of obesity and chronic diseases (2). In adults, plant-based diets have shown significant health improvements and reductions in medication use. This underscores their feasibility in promoting community health (8). Nutrition is also crucial for disease prevention and management, as demonstrated in studies that link low-inflammatory diets to reduced symptoms in arthritis patients (3), while other dietary strategies, such as time-restricted eating, have shown promise in enhancing metabolic health and in supporting circadian rhythms (6). Physical activity further bolsters health, with studies noting its benefits on mental health (14) and resilience through the release of bioactive molecules called exerkines. These molecules aid muscle growth and metabolic regulation (9). Exercise also supports cardiovascular health (13), aids in the prevention of musculoskeletal decline (12), and improves mental health outcomes in adults with spinal cord injuries (16).

Sleep quality is similarly essential, with poor sleep linked to cardiovascular morbidity, metabolic issues, and mental health risks (18, 20). Irregular sleep patterns have significant health impacts, including contributing to multimorbidity among military personnel (22) and mental health issues in adolescents, particularly during the COVID-19 pandemic (21). Sleep hygiene improvements, such as promoting regular sleep schedules, are recommended to combat these effects (17). In fact, stress and sleep health are interrelated, influencing mental health, mood, and resilience, with regular physical activity and a balanced diet offering protective effects against stress-related health issues (4, 11). Together, these lifestyle factors not only shape individual health but also serve as cornerstones for preventive health measures and holistic wellness across different life stages.

### 3.2 Effects of environment on individual health

Environmental factors, such as air quality, pollution, and climate, significantly impact individual health by contributing to a range of physical and mental health issues. Poor air quality is a notable risk factor for cardiovascular and respiratory diseases, with pollutants like particulate matter, nitrogen dioxide, and ozone leading to inflammation and oxidative stress (24, 27). Indoor air pollution also poses serious health risks in urban environments, particularly in rapidly industrializing regions (28). Innovative solutions like portable air purification can mitigate these effects (23). Climate change compounds these issues, with extreme weather events and increased UV exposure intensifying health risks. Coates et al. (29) and Ebi et al. (30) underscore the effects of climate variability, which aggravates dermatological and heat-related illnesses, especially in vulnerable populations. Furthermore, water pollution remains a significant health threat, with contaminants like lead and PFAS (per- and polyfluoroalkyl substances) affecting vulnerable groups (43, 45). Liu et al. (52) and Sharma et al. (46) highlight that untreated sewage and chemical pollution in freshwater sources lead to gastrointestinal and other waterborne diseases. The mental health implications of climate change are also crucial, as extreme weather and pollution contribute to increased psychological distress, particularly among marginalized communities (34). Public health strategies that address these environmental determinants, such as improved health literacy (32) and localized pollution assessments (25), are essential. Interdisciplinary approaches that integrate pollution control with health interventions, as advocated by Xu et al. (41), are vital for reducing the overall burden of environmental health risks and supporting resilience against climate change's impacts on human health.

### 3.3 The need for continuous and reliable data collection to personalize care effectively

Continuous and reliable data collection is essential for effective personalization in healthcare, as it enables real-time insights into the evolving health status of a patient. Traditional episodic health check-ups capture only snapshots of the health of an individual, often missing critical daily, hourly, minute-by-minute, or even second-by-second fluctuations in factors like blood glucose, heart rate, activity levels, and environmental exposures. With the integration of wearable IoT devices, advanced sensors, and high-speed 6G connectivity, healthcare providers will be able to collect and analyze ongoing data streams, in order to build comprehensive pictures of the unique needs of each patient. This allows for hyper-personalized adjustments to treatment plans based on real-time conditions. This will facilitate preventive care, early interventions, and targeted therapies. Reliable, continuous data collection thus transforms care from reactive to proactive. This supports individualized health strategies that improve long-term outcomes and quality of life.

In this context, reinforcement learning (68) and recommender systems (69, 70) can play a vital role. Reinforcement learning algorithms can adaptively adjust treatment plans by learning from new data and optimizing health strategies based on physiological responses and patient feedback over time. Similarly, recommender systems use ongoing data to offer hyper-personalized suggestions, such as dietary changes or physical activity adjustments, which can be aligned with treatment plans, individual health goals, and lifestyle. Together, these technologies enable a dynamic, proactive approach to healthcare that supports long-term health and quality of life improvements.

## 4 Quantum computing and AGI in hyper-personalized medicine

### 4.1 Definitions

Quantum computing, based on the principles of quantum mechanics, will provide unmatched computational abilities through quantum-speed processing. By enabling massive parallel processing, quantum computing can analyze extensive and complex datasets that classical computers cannot handle effectively (71). This technology is well-suited for healthcare applications, particularly in hyper-personalized medicine, where customized treatments require detailed biological and environmental data (72).

Artificial General Intelligence (AGI), more advanced than conventional AI, will offer adaptable learning across various tasks, which enables it to interpret and adjust recommendations for a wide range of data, including health metrics, environmental factors, and lifestyle information (73). The responsive and flexible learning of AGI will allow it to create dynamic treatment plans that continuously integrate new data. This will make it especially valuable for hyper-personalized healthcare.

Hyper-personalized medicine aims to deliver highly individualized healthcare by incorporating genetic, lifestyle, and environmental data. Using quantum computing and AGI as its technological foundations, hyper-personalized medicine will adapt diagnosis and treatment more accurately to patient needs, thus improving outcomes (74, 75).

### 4.2 Data processing, prediction, and modeling

The processing power of quantum computing will transform data analysis for hyper-personalized medicine and will analyze vast datasets, such as genomic and environmental data, with unmatched speed and accuracy. By enabling high-precision and real-time data analysis, quantum computing will uncover patterns and risks that were previously undetectable (72, 76). Quantum-enabled data processing will also support the predictive abilities of AGI and will allow it to customize treatment plans based on insights drawn from real-time health changes. With this capacity, AGI can provide personalized recommendations that continuously adjust in response to updated biometrics, dietary changes, stress levels, and environmental exposures, thus leading to highly effective and tailored care (71).

Quantum computing will also support AGI in developing detailed simulations and predictive models for individual health scenarios. Quantum simulations will map molecular and cellular interactions and predict the effects of specific treatments on biological pathways. By modeling environmental and lifestyle interactions with genetic data, quantum computing will provide AGI with insights that allow for continuously refined recommendations based on changes in pollution levels, diet, or activity (74, 76). These simulations will guide AGI in predicting disease progression and in determining the most effective treatments with minimal trial and error. These will reduce the need for real-world trials and make hyper-personalized care more accessible and affordable (77).

## 5 IoT and 6G: real-time data collection and connectivity

### 5.1 Role of IoT and 6G in data collection

IoT sensors monitor lifestyle and environmental factors that influence health, which will support hyper-personalized medicine by continuously gathering data on health metrics like heart rate, blood pressure, and activity levels. Environmental sensors measure air quality, temperature, and humidity, which allow healthcare providers to understand how surroundings impact health outcomes (78). These sensors will adapt data collection methods based on patient location and activities to provide AGI with context-specific data. AGI will use this information to create health recommendations that align with a patient's immediate environment and daily routines. This will enhance the precision of care (72).

With 6G networks, data transmission will be instantaneous and will facilitate seamless communication between IoT devices and healthcare systems. The ultra-fast data rates and low latency of 6G will support real-time adjustments in treatment plans by ensuring immediate access to health metrics and environmental data (73, 79). Additionally, enhanced bandwidth and scalability will allow a broad network of IoT-connected healthcare devices to operate in both urban and rural areas, and will thus, expand access to real-time monitoring. The advanced encryption and privacy protections of 6G promises to secure sensitive health data during transmission and will ensure that data remain protected while enabling rapid and responsive healthcare interventions (80).

### 5.2 Enhanced precision and context-aware care

The integration of 6G with IoT will bring an unprecedented level of precision to AGI-driven healthcare. With continuous real-time updates, AGI will dynamically adjust care recommendations and reduce errors to ensure that treatment is responsive to the patient's health needs (79, 81). This high-precision data will support individualized interventions by allowing AGI to adapt care based on factors such as activity levels, medication, and environmental conditions. By integrating data from multiple IoT sources into a single health profile, AGI will ensure a consistent and accurate understanding of the patient's health and thus, increase transparency and trust in the healthcare process (82).

Context-aware care will further enhance personalized healthcare by adjusting AGI recommendations based on environmental factors like air quality or pollution levels. AGI will use environmental data to provide timely interventions, such as advising patients to avoid specific areas on high-pollution days or adjusting medication based on current conditions (77). The ability to access live public health advisories will ensure that AGI recommendations align with a patient's environment, creating a holistic approach to hyper-personalized medicine that addresses both individual health and broader public health considerations (73).

## 6 Practical applications and benefits of hyper-personalized medicine

### 6.1 Proactive health interventions and monitoring

With the support of 6G connectivity, AGI will enable proactive health interventions by adjusting treatment plans in real time, responding to any sudden changes in the patient's health. This capability will allow AGI-driven systems to provide prompt recommendations on diet, physical activity, and lifestyle adjustments, aligning them with biometrics and environmental factors to support optimal patient health (81). Furthermore, health alerts issued through AGI will help patients take preventive actions, such as staying hydrated during extreme heat or modifying exercise routines based on current air quality (78). This proactive approach will enhance patient wellness and reduce potential health risks.

Continuous monitoring through IoT-enabled wearables will support the efforts of AGI in tracking vital health metrics like heart rate, glucose levels, and sleep patterns. As AGI detects any deviations from a patient's normal data, it will initiate timely interventions that address emerging health issues before they escalate (82). Patients will access these insights through health dashboards, which will provide ongoing metric updates and empower individuals to engage actively in their health management.

Real-time glucose monitoring has been significantly enhanced by advancements in wearable technologies. For instance, non-invasive sensors are being developed to monitor glucose levels through biofluids like sweat, saliva, and interstitial fluid, leveraging their correlation with blood glucose concentrations. However, challenges such as accuracy, lag time, and the seamless integration of data into electronic health records persist. The incorporation of Internet of Things (IoT) networks can address integration issues by enabling direct connectivity between sensors and healthcare platforms. Additionally, emerging 6G technology, with its unprecedented speed of up to 1 terabyte per second, can reduce data latency and enable truly real-time monitoring. These systems can achieve accuracy akin to clinical standards when powered by AGI algorithms and quantum computing, fostering the potential for hyper-personalized diabetes management through continuous feedback and predictive analytics (83).

Personalized diet and exercise plans are another frontier for proactive health interventions. Current AI-powered diet planning systems often lack scalability and interpretability, hindering widespread application. The proposed *Healthcare 5.0* framework, which combines IoT, 6G, AGI, and quantum computing, can be used to tackle these issues. IoT can enable the integration of various wearable and environmental sensors into cohesive networks, while 6G will ensure the rapid processing of vast datasets. Quantum computing will provide the computational power required to analyze complex relationships between genetic, dietary, and physical activity data, facilitating individualized recommendations. AGI will enhance system interpretability by identifying actionable insights from multivariate datasets, bridging the gap between machine-generated plans and human-understandable guidance (84).

Virtual healthcare assistants (VHAs) demonstrate the transformative potential of digital health technologies. Within the *Healthcare 5.0* framework, VHAs will utilize IoT-connected wearable sensors to collect and analyze comprehensive health data. With the rapid connectivity of 6G, these systems will provide real-time adaptive health recommendations tailored to users' evolving needs. By integrating AGI, VHAs can effectively interpret user data and offer contextually relevant advice, ensuring empathetic and precise interactions. For example, they might provide timely reminders for hydration based on sweat sensor data or suggest exercises compatible with an individual's metabolic profile. Furthermore, the reliance of VHAs on quantum computing ensures robust data processing, supporting predictive analytics to anticipate health risks and recommend preemptive measures (85).

### 6.2 Contextualized health support

Through the integration of IoT and 6G, AGI will provide context-sensitive health support by tailoring care based on real-time environmental conditions. This capability will allow AGI to make specific recommendations, such as advising patients with respiratory conditions to avoid outdoor activities on days with poor air quality or adjusting medications to account for seasonal allergies (86). The access of AGI to environmental data will enable it to issue timely alerts, which will help patients to make informed decisions and avoid exposure to health risks associated with local conditions. IoT devices will also incorporate public health advisories in order to ensure that the recommendations made by AGI align with community health guidelines and environmental data to deliver holistic and personalized care (73).

## 7 Challenges and ethical considerations

### 7.1 Privacy and security

The vast amounts of personal data generated by IoT devices and real-time health monitoring raise significant privacy concerns, as personal health data is among the most sensitive information collected. Its gathering, storage, and transmission through IoT and 6G networks could lead to severe privacy violations if not managed with the highest security standards. To mitigate these risks, a decentralized approach to data storage, aligned with deontological ethics, is essential. Deontological ethics (87–90), which emphasizes the moral duty to protect individual rights, highlights the ethical obligation to uphold privacy rigorously. This responsibility entails implementing strong encryption and anonymization protocols, along with giving users control over their data access, ensuring respect for individuals' autonomy. By addressing privacy through this ethical framework, healthcare providers, network providers, and developers fulfill their ethical obligations to safeguard personal health data by emphasizing a commitment to the inherent rights of each individual to confidentiality and data protection.

Bouderhem explored issues related to AI ethics and proposes several practical solutions to address them (91). The main challenges of AI ethics span a wide range of issues, including safeguarding data privacy, ensuring the secure collection and storage of data, and addressing concerns about data quality, availability, and accuracy. Interoperability between different operating systems, such as iOS and Android adds further complexity, while issues like bias, health equity, fairness, and the affordability and accessibility of AI in developing countries present significant obstacles. Additional challenges include the regulation and governance of AI systems, controlling third-party access to personal health data, and maintaining robust security. Successful implementation and adoption of AI require solutions to problems such as lack of explainability, lack of transparency, lack of accountability, errors, misdiagnosis, discrimination, and poor overall system performance.

To ensure the privacy and security of personal health data, measures such as educating healthcare personnel, conducting routine risk assessments, securing data with VPNs, and restricting access to sensitive information are essential. Role-based access control, two-factor authentication, data encryption, and security awareness training further enhance protection against breaches. Regulating AI systems effectively requires a comprehensive approach that includes establishing legally binding rules and standards under the guidance of the World Health Organization (WHO), strengthening regulatory oversight, and promoting transparency and accountability. Encouraging industry self-regulation, fostering international cooperation, embedding ethical practices in handling personal health data, and creating a collaborative “AI culture” involving all stakeholders are vital steps toward achieving responsible and effective AI governance.

A critical challenge in AI ethics in healthcare is bias. Bias arises when predictive models perform unevenly across different demographic or clinical subgroups, often due to imbalanced or unrepresentative data. This can result in disparities in diagnosis, treatment recommendations, or outcomes, disproportionately affecting underrepresented or vulnerable populations. Factors contributing to bias include data imbalance, where certain groups, such as ethnic minorities or the older adults, are underrepresented in training datasets, and systemic bias, where historical inequities in healthcare practices are inadvertently perpetuated by AI systems. The consequences of bias are significant, as they can exacerbate health disparities and undermine trust in AI-driven healthcare solutions, such as the *Healthcare 5.0* framework.

To address these challenges, several strategies can be implemented. First, ensuring datasets are diverse and representative of all relevant subgroups is critical, with efforts focused on collecting data from varied populations, augmenting underrepresented classes using synthetic data, and regularly auditing datasets for bias. Second, bias detection and mitigation techniques, such as fairness metrics, reweighting, and adversarial debiasing, should be integrated into the development process to identify and correct disparities. Third, continuous learning AI systems can help overcome bias by updating models with new, real-world data and incorporating feedback loops to adapt to changing population characteristics (92). These systems can also employ federated learning to enhance representation while maintaining privacy. Therefore, the *Healthcare 5.0* adopted system feedback for system improvement and addressing bias.

Transparent and explainable AI (XAI) techniques, along with Artificial General Intelligence (AGI), will become essential for identifying and addressing biases in healthcare AI systems. These technologies will enable healthcare professionals to interpret predictions and make informed decisions. Transparency fosters accountability and builds trust, particularly in high-stakes applications like diagnostics and treatment planning. Moreover, ethical and regulatory oversight is vital, with governments and organizations establishing guidelines to ensure fairness, such as mandating fairness audits and documenting data sources and evaluation criteria. Addressing bias in healthcare AI is both a technical challenge and a moral imperative. By adopting these strategies, the *Healthcare 5.0* framework can develop equitable and reliable healthcare solutions that benefit all populations.

The integration of advanced sensors, IoT, and AI provides many advantages in healthcare. However, the massive volumes of data collected by these sensors, along with health records and existing big data on networks, can create an overload of data, leading to bottlenecks, especially in real-time monitoring within the *Healthcare 5.0* framework (93). To address these challenges, edge computing can be deployed near data sources to enable real-time analysis and reduce reliance on centralized servers. For example, edge AI can detect abnormal vital signs and send immediate alerts, minimizing the need to transmit raw data (94). A hybrid approach combining edge and cloud computing can address both real-time and long-term data processing needs. Critical data can be processed locally on edge devices, while less urgent information can be transmitted to the cloud for deeper analytics and archival purposes.

Data fusion techniques can further reduce redundancy by consolidating information from various sources, such as wearable devices, imaging systems, and patient records, to create comprehensive datasets for more accurate diagnostics. AI-driven network optimization can dynamically manage data traffic, predict congestion, and reallocate bandwidth to ensure smooth transmission of high-priority healthcare data. Efficient data compression and prioritization algorithms are also crucial for overcoming bottlenecks by reducing the size of transmitted data and ensuring that critical information is processed first (95). Standardizing data formats and communication protocols, such as adopting HL7 FHIR, can enhance interoperability and streamline data exchange, mitigating delays caused by incompatible systems. Moreover, blockchain technology can offer a secure and decentralized method for data sharing across stakeholders, eliminating intermediaries and maintaining data integrity in multi-institutional collaborations or telehealth systems.

### 7.2 Accessibility and health equity

The reliance on advanced IoT and 6G-powered healthcare solutions could pose potential risks to health equity, as those in economically disadvantaged or rural areas may lack access to these innovations. In fact, limited infrastructure and access to technology has been leaving large populations, especially in low- and middle-income countries without essential health services (96). Without equitable access, entire populations may miss out on the benefits of hyper-personalized medicine. This would exacerbate existing health disparities. From a utilitarian ethics perspective (97–99), which seeks to maximize societal benefit and reduce harm, healthcare must strive for inclusivity in technology distribution. This ethical approach underscores the need for policies promoting equitable access, in order to ensure that the positive impact of healthcare reaches the greatest number of people. Policymakers and healthcare providers must therefore focus on creating inclusive strategies, such as subsidized devices and scalable network infrastructure for underserved areas, and encourage inclusive design practices to support a fair distribution of healthcare benefits, thereby minimizing the risk of creating a healthcare divide.

### 7.3 System reliability

For hyper-personalized medicine to be effective, system reliability in continuous, real-time healthcare is critical. Breaks in 6G connectivity or device malfunctions can disrupt data flow, and thus, potentially delay crucial health interventions or lead to misinformed treatment changes. From an ethics of care perspective (100–102), which emphasizes the moral responsibility to care for others' wellbeing, ensuring system reliability is central to building a trustworthy healthcare environment. This ethical framework prioritizes accountability and responsiveness and calls on network providers and device manufacturers to establish fail-safe mechanisms, backup connectivity options, and rigorous quality control measures. By upholding these standards, providers demonstrate a commitment to patient welfare and ensure that hyper-personalized medicine remains a reliable and safe resource. This approach not only safeguards health outcomes but also strengthens patient trust in technology-dependent healthcare.

### 7.4 Doctor-patient dynamics and socio-behavioral implications in hyper-personalized medicine

The advent of hyper-personalized medicine, driven by advancements in genomics, artificial intelligence (AI), and data analytics, will fundamentally reshape the doctor-patient relationship. Traditionally, doctors have been the primary decision-makers, guiding patients based on standardized protocols and evidence-based practices. In hyper-personalized medicine, the dynamic will shift toward shared decision-making, with patients actively participating as their genetic, lifestyle, and environmental data inform tailored treatment options. This evolution not only empowers patients but also requires physicians to develop advanced communication skills to interpret and convey complex datasets effectively. The socio-behavioral implication in hyper-personalized medicine is similar to the one of precision medicine (PM). Eyal et al. (103) provide summary of the physician-patient relationship in the age of PM that can be the picture of how the doctor-patient interaction in hyper-personalized medicine. Traditionally, the doctor-patient relationship follows a simplified framework where being “sick” is viewed as a distinct social role. Society assigns this role during doctor-patient interactions, aided by diagnostic tools and expert heuristics that categorize individuals as either “healthy” or “sick.” This “sick role” grants individuals' exemption from societal obligations, provided they actively seek help from qualified medical professionals. It anchors patients in a social exchange of trust and care, with the shared goal of restoring normal functioning. Physicians, in this model, act as gatekeepers, determining access to the “sick” status, diagnoses, and the limited resources within the healthcare system. Their authority is rooted in the voluntary nature of the sick role, medicine's ethical standards, and the inherent knowledge gap between doctors and patients.

Personalized medicine and precision medicine (PM), and also hyper-personalized medicine, disrupt the binary “healthy or sick” framework, replacing it with a spectrum of hybrid statuses. Patients are categorized based on genetic, environmental, and behavioral data, leading to roles like “patients-in-waiting,” where individuals may be monitored or treated prophylactically despite uncertain diagnoses. This creates ambiguity for patients and their families, as traditional healthcare scripts fail to provide clear answers. For example, families often struggle with the unclear implications of genetic screening results, leading to stress and confusion as they navigate unfamiliar and unstable healthcare scenarios.

Hyper-personalized medicine also intensifies uncertainty, shifting the psychological burden onto patients. Probabilistic diagnoses, while intended to empower patients through informed decision-making, can paradoxically increase stress. Socioeconomic, cultural, and psychological disparities further exacerbate these challenges, leaving many patients ill-equipped to manage the complexities of their care. Additionally, patients must increasingly surrender their privacy as healthcare systems collect detailed data on genetic, environmental, and behavioral factors. Through active tracking and passive monitoring, individuals lose control over their information and how it is used, raising concerns about data privacy and ethical boundaries.

Doctors will face new responsibilities as hyper-personalized medicine transforms their role from managing patient complaints to performing “bridging work.” This involves reconciling test results with patients' symptoms, or lack thereof, and addressing discrepancies in diagnoses. Doctors may redefine treatment as prevention or adjust diagnostic thresholds, but many lack the genomic expertise and resources needed for these tasks. The uncertainty surrounding interactions between environmental, lifestyle, and genetic factors further complicates their role.

New knowledge asymmetries will emerge as hyper-personalized medicine advances. Doctors may struggle to interpret genomic data and manage large-scale datasets, shifting informational dominance to specialists, labs, and platform managers. To address this gap and prevent inequities, especially in underserved settings, doctors must receive specialized training to mediate and disclose hyper-personalized medicine findings effectively. Without such adaptations, hyper-personalized medicine findings risks deepening existing disparities in healthcare access and outcomes. Hyper-personalized medicine transforms traditional roles in the healthcare system, introducing uncertainty, redistributing authority, and reshaping the doctor-patient dynamic. These changes demand significant adjustments from all stakeholders to navigate the complexities of hyper personalized medicine.

## 8 Discussion: a vision for the future

Integrating quantum computing, AGI, IoT, and 6G into a comprehensive healthcare framework can revolutionize medicine by creating a model that addresses the nuanced interplay between lifestyle, environmental influences, and genetics. This integration is fundamental for hyper-personalized healthcare, where understanding each individual's unique combination of genetic, lifestyle, and environmental factors will enable precise and responsive treatment. Unlike traditional healthcare, which often relies on static and generalized treatment protocols, this model adapts dynamically to real-time data, drawing insights from a wealth of patient-specific information. By continuously monitoring variables like biometrics, daily activities, air quality, and stress levels, hyper-personalized medicine can provide recommendations that respond to changes as they occur. The convergence of these technologies within an advanced *Healthcare 5.0* framework will build a responsive and patient-centered model, one that prioritizes both human wellbeing and resilience in a rapidly changing world.

Quantum computing will play a vital role in handling the extensive and complex data that personalized medicine requires. With its ability to process massive datasets quickly and accurately, quantum computing will enable the development of predictive models that account for the intricate interactions between genes, environment, and behavior. This computing power will allow AGI to predict potential health outcomes and adjust treatment plans dynamically. This will result in a healthcare system that can proactively address health risks and optimize patient care. Unlike classical AI, AGI will be capable of learning and adapting across diverse data types and health domains and will facilitate a model that not only makes predictions but also adapts as new data becomes available. The flexible learning of AGI, coupled with quantum-enhanced analytics, will provide a foundation for continuously evolving healthcare insights, ensuring that patient care remains relevant and responsive to their unique ongoing needs.

The IoT and 6G components will act as the backbone for real-time data collection and connectivity within this framework. IoT devices, from wearable fitness trackers to environmental sensors, will capture a constant stream of data in order to offer a holistic view of the patient's health and surroundings. These devices will monitor essential health metrics, environmental factors, and behavioral data, which will inform AGI-driven recommendations and provide actionable insights for both patients and providers. With 6G connectivity, these data streams will be transmitted instantly. Delays will be eliminated and real-time intervention will be enabled. This connectivity will ensure that patients receive prompt alerts and recommendations, whether they need to adjust a medication dosage, avoid a particular location due to high pollution, or hydrate during a heatwave. The high-speed, low-latency capabilities of 6G will make these instantaneous responses possible and will create a seamless flow of information that enhances patient safety and care quality.

To make this vision a reality, robust policy and infrastructure development are essential to support widespread access to hyper-personalized, IoT-enabled healthcare. Policymakers must focus on creating an inclusive digital infrastructure that enables access to IoT devices and 6G connectivity across socioeconomic backgrounds and geographic regions. Investment in public infrastructure will be crucial, particularly in underserved rural and urban areas where limited access to technology could create disparities in healthcare access. By prioritizing the deployment of these technologies in underserved communities, governments and private entities can work together to ensure that hyper-personalized healthcare is accessible to all, rather than an exclusive privilege for those in well-resourced regions. Subsidies for IoT devices and healthcare services could be another viable option to bridge the gap in access, making it feasible for low-income individuals to benefit from these advancements in healthcare technology.

Developing this framework also requires collaboration between healthcare providers, technologists, and regulatory bodies to address ethical and practical challenges associated with hyper-personalized medicine. Privacy and security concerns, for example, must be at the forefront of policy development, as IoT and 6G networks generate extensive personal health data that must be protected rigorously. Decentralized storage solutions, robust encryption, and user control over data access can help mitigate privacy risks, allowing patients to participate in data sharing with confidence. Additionally, establishing ethical guidelines for data use and access is essential to building a trustworthy healthcare system, where patients feel that their personal information is handled with integrity. Regulatory bodies will need to establish clear policies that enforce these standards, ensuring that all stakeholders prioritize patient privacy and security in the design and implementation of hyper-personalized healthcare technologies.

To realize a truly equitable and effective *Healthcare 5.0* model, a focus on human-centered care and resilience is necessary. Hyper-personalized medicine inherently aligns with the goals of the European Commission's *Industry 5.0* framework, which emphasizes sustainability, human-centric approaches, and adaptability. *Healthcare 5.0*, as a parallel framework, would build on these principles, prioritizing not only individualized care but also environmental consciousness and the capacity to adapt to global health challenges. By utilizing resources responsibly and minimizing waste, *Healthcare 5.0* can maintain a focus on sustainability, addressing the health of the planet alongside the health of the individual. This approach acknowledges that human health is inextricably linked to environmental health, and by minimizing the environmental impact of healthcare technologies, we can promote a system that supports both planetary and personal resilience.

Promoting health equity is a key component of *Healthcare 5.0*, as the risks of exacerbating health disparities through advanced technologies must be acknowledged and addressed. For hyper-personalized medicine to be universally beneficial, policymakers and healthcare providers need to ensure that the advantages of IoT, 6G, quantum computing, and AGI reach underserved populations. Affordable and accessible healthcare solutions, powered by these technologies, should become a priority for public health initiatives. Additionally, implementing inclusive technology design, such as multilingual support and intuitive interfaces, will enable broader participation in hyper-personalized healthcare, ensuring that individuals of all backgrounds and abilities can benefit from these innovations. Policymakers must advocate for equitable resource allocation and accessible infrastructure to minimize the healthcare divide and maximize the societal benefits of *Healthcare 5.0*.

To achieve this vision, a collaborative approach is essential. Policymakers, healthcare providers, researchers, and technology developers need to come together to align their efforts, working within a shared framework of objectives and ethical guidelines. Interdisciplinary partnerships can drive innovation, creating a healthcare system that integrates emerging technologies into a cohesive and human-centered model. Educational initiatives can also play a role in supporting this transition, as healthcare providers and patients alike will need to understand how to engage with these new technologies effectively. Training programs for healthcare professionals in AGI, quantum computing, IoT, and 6G will be crucial to ensure that they can use these tools to their full potential, while public health campaigns can raise awareness about the benefits and responsibilities of hyper-personalized healthcare.

### 8.1 How do we get there?

Achieving this advanced model of healthcare requires clear action steps from each stakeholder involved. First, governments must prioritize digital infrastructure investments to lay the groundwork for widespread IoT and 6G deployment, especially in under-resourced areas. Healthcare institutions and technology companies should work collaboratively to develop secure data-sharing frameworks that protect patient privacy while allowing the necessary data flow for personalized care. Research and development in quantum computing and AGI must also continue at a rapid pace, focusing on scalable, cost-effective applications that align with the needs of the healthcare sector.

Meanwhile, healthcare providers should begin integrating IoT-enabled devices into their practices, familiarizing themselves with data from wearables and environmental sensors to create more comprehensive patient profiles. Educational institutions should incorporate AGI, IoT, and quantum computing concepts into medical training programs, preparing future healthcare professionals to operate in a hyper-personalized, technology-driven environment. Developing partnerships with tech companies and research institutions will accelerate this process, as interdisciplinary collaboration is essential to creating a healthcare system that integrates cutting-edge technologies seamlessly.

A crucial next step involves engaging patients and the public. Effective communication about the benefits of hyper-personalized medicine, as well as transparent explanations of data use and security, can foster trust and willingness to participate in this healthcare model. Public health campaigns and community workshops can demystify these technologies, addressing potential concerns and showcasing the ways hyper-personalized care can enhance quality of life. Such engagement is particularly important for marginalized communities that may have historically experienced healthcare disparities.

### 8.2 A call to action

In conclusion, hyper-personalized medicine has the potential to revolutionize healthcare by integrating quantum computing, AGI, IoT, and 6G into a comprehensive and human-centered model. However, realizing this vision requires more than technological advancement; it demands a commitment to equity, ethical responsibility, and resilience. We call on all stakeholders, from policymakers and healthcare providers to technologists and patients, to contribute to building a healthcare system that prioritizes individual wellbeing, environmental sustainability, and universal access. This endeavor represents a collective effort, where each sector's actions influence the development and success of hyper-personalized medicine.

By aligning our goals with the *Healthcare 5.0* framework, we can ensure that the innovations we create not only enhance individual care but also foster a sustainable and resilient healthcare infrastructure. Let us seize this opportunity to redefine healthcare, transforming it into a system that supports the health of every individual while addressing the broader challenges of our global environment. Together, we can pave the way toward a future where healthcare is truly personalized, equitable, and prepared to meet the needs of a changing world.
